# Population-level evidence on lifestyle-related education and preventive services in primary care in Poland—a 2025 cross-sectional survey

**DOI:** 10.3389/fpubh.2026.1779567

**Published:** 2026-03-27

**Authors:** Justyna Grudziąż-Sękowska, Kuba Sękowski, Mateusz Jankowski, Stanisław Surma, Agnieszka Kamińska, Agata Olearczyk

**Affiliations:** 1 School of Public Health, Centre of Postgraduate Medical Education, Warsaw, Poland; 2 Department of Internal Medicine and Clinical Pharmacology, Medical University of Silesia, Katowice, Poland; 3 Faculty of Medicine, Collegium Medicum, Cardinal Stefan Wyszynski University, Warsaw, Poland; 4 Health Innovation Unit, SGH Warsaw School of Economics, Warsaw, Poland

**Keywords:** family medicine, health education, health prevention, Poland, primary care, public health, screening

## Abstract

**Introduction:**

Primary health care serves as the patient’s first point of contact with the healthcare system and plays an important role in prevention and health education. This study aimed to assess public perceptions of lifestyle-related education and preventive services provided by primary health care in Poland, as well as identify factors associated with willingness to use educational and preventive services offered in primary care.

**Methods:**

This cross-sectional survey was carried out in October 2025 in a nationwide sample of 1,090 adults in Poland. Quota sampling was used. Data were collected through computer-assisted web interviews (CAWI).

**Results:**

Among the respondents, 67.3% (*n* = 734) had visited a primary care physician due to their own health status at least once in the last 12 months. Of those, 53% declared having received at least one healthy lifestyle-related advice from the primary care staff. Preventive tests appropriate for the patient’s age (33.2%) were the most common, followed by principles of healthy eating (19.8%) and physical activity (17.4%). In the multivariable logistic regression model, female gender (aOR: 1.72 [1.28–2.32], *p* < 0.001) and higher education (aOR: 1.38 [1.03–1.86]; *p* < 0.03) were significantly associated with lower odds of receiving lifestyle-related advice. Among all respondents, 67.8% declared a willingness to receive an annual individualized prevention plan from their primary care physician. Being married (aOR: 1.46 [1.07–1.98]; *p* = 0.02), and visiting a primary care physician (at least once) in the last 12 months (aOR: 2.66 [2.03–3.49]; *p* < 0.001) were significantly associated with willingness to receive an annual individualized prevention plan.

**Conclusion:**

This study showed a low level of implementation of lifestyle-related education and preventive services in primary care in Poland. There is a public expectation towards the implementation of new preventive services in primary care, like an annual individualized prevention plan.

## 1 Introduction

Primary health care (PHC) constitutes a core component of the healthcare system in Poland, alongside outpatient specialist care and hospital services (1, 2). It serves as the patient’s first point of contact with the healthcare system and plays a key role in ensuring continuity of care and coordination of diagnostic and therapeutic procedures (1). The organizational framework of PHC is regulated by national legislation and is based on teamwork involving physicians, nurses, midwives, and support staff (2). This team-based structure creates a potential setting not only for the diagnosis and treatment of diseases but also for the implementation of preventive and educational activities throughout the life course (1–3).

Contemporary public health challenges are largely driven by chronic non-communicable diseases and their behavioral determinants (3). Lifestyle-related factors such as unhealthy diet, insufficient physical activity, tobacco use, and harmful alcohol consumption substantially contribute to cardiovascular, metabolic, and oncological risk, leading to a significant burden of disease and healthcare costs (4). Effective prevention therefore requires actions that are implemented early, systematically, and in a setting that is easily accessible to the population (5). International organizations emphasize the importance of integrating prevention and health promotion into routine primary care practice, as primary care providers are uniquely positioned to reach patients before the onset of advanced disease (6, 7). Comparative studies further indicate that strong primary health care—characterized by accessibility, continuity, coordination, and comprehensiveness—is associated with improved population health outcomes and greater efficiency of healthcare systems (7, 8).

Preventive activities in primary health care encompass a broad range of services, including referrals for diagnostic tests, support for vaccinations and population-based screening programs, as well as health education and lifestyle counseling. In recent years, Polish health policy has increasingly emphasized strengthening the preventive role of PHC. This shift is reflected in the introduction of coordinated care models in primary care, which aim to expand diagnostic and therapeutic pathways for selected conditions while promoting collaboration within the primary care team (9). Educational counseling is an integral element of these models and is formally recognized in national guidelines and reimbursement mechanisms implemented by the National Health Fund (10). In Poland, preventive activities in primary care are financed through the National Health Fund (NFZ), including preventive programs and coordinated care models that incorporate elements of health education and lifestyle counseling (10).

Despite these systemic developments, available evidence suggests that the preventive and educational potential of primary health care in Poland is not fully utilized in routine clinical practice (11). Primary care services remain predominantly associated with the management of acute conditions and the control of existing chronic diseases rather than with proactive prevention (11). This is particularly noteworthy given that Polish patients highly value the accessibility of primary care services and the opportunity to establish long-term relationships with their primary care physicians, which may facilitate trust and support behavioral change (12, 13). Data from the European EUROPREV (European Network for Prevention and Health Promotion in Family Medicine and General Practice) study indicate that a substantial proportion of primary care patients do not report discussing key lifestyle-related topics—such as diet, physical activity, or smoking—with their physicians, even when relevant risk factors are present (14).

In parallel with organizational reforms, new preventive initiatives targeting the adult population have recently been introduced in Poland. One example is the “Moje Zdrowie” program, implemented in primary care settings, which combines risk assessment, diagnostic testing, and the development of individualized health plans (15). Additionally, increasing attention is being paid to the role of preventive medicine specialists, whose professional responsibilities focus on planning and delivering preventive and educational activities within the healthcare system and public health structures (11, 16). These developments suggest a growing recognition of prevention as a core function of primary health care. Still, despite clear guidelines, educational programs, training, etc., the prevalence of classic cardiovascular risk factors in Poland has not changed significantly for years (17).

However, population-based data assessing the actual delivery of lifestyle-related education and preventive counseling in routine primary care practice in Poland remain limited (17). Administrative and service-level data do not capture patient experiences or the content of preventive counseling provided during routine consultations, which limits the ability to evaluate how preventive recommendations are implemented in everyday primary care practice (11). This lack of evidence hampers informed public health planning and the identification of population groups that may be underserved by preventive services. To date, no nationwide population-based study has comprehensively examined patient-reported experiences of lifestyle-related education and preventive services delivered in routine primary health care in Poland.

Therefore, we conducted a population-based study aimed at assessing public perceptions of lifestyle-related education and preventive services provided by primary health care in Poland, as well as identifying factors associated with willingness to use educational and preventive services offered in primary care. We hypothesized that the implementation of lifestyle-related education in primary care remains limited and that socio-demographic factors may influence both patient experiences of preventive counseling and willingness to receive preventive services. The findings of this study are intended to support future organizational and policy decisions aimed at strengthening the preventive role of primary health care.

## 2 Materials and methods

### 2.1 Study design and data collection

A nationwide, cross-sectional survey of adults (≥18 years) living in Poland was conducted between 10 and 13 October 2025. The target population was the general adult population. Data were collected using Computer-Assisted Web Interviewing (CAWI). A professional polling company [Nationwide Research Panel Ariadna (18)] was contracted to collect data following the methodology and study design prepared by the authors. Procedures were consistent with earlier population-based surveys in Poland (19–21).

Respondents were sampled from a registry exceeding 100,000 unique, verified adults. Quota sampling with stratification for sex, age group, and place of residence was applied. Quotas were adjusted to the national demographic profile (gender, age, size of the place of residence) using Statistics Poland reports (22). The minimal sample size calculation was performed based on Statistics Poland reports (22).

Individualized invitations (SMS and e-mail) containing a secure survey link hosted on the polling company web platform (18) were issued to sampled panelists. Non-responders were replaced by panelists meeting the same strata until quotas were satisfied. The questionnaire enforced complete responses; therefore, there was no missing data. In total, 1,090 fully completed questionnaires were obtained. Estimated response rate of the Nationwide Research Panel Ariadna is 22%.

The study protocol received approval from the Ethics Committee at the Centre of Postgraduate Medical Education (decision No. 88/2025; 8 October 2025). All procedures followed the Declaration of Helsinki. Participation was voluntary and anonymous. Informed consents were collected.

### 2.2 Questionnaire development and measures

The study questionnaire was self-prepared, based on the literature review (3–12). The questionnaire included 12 questions on primary care services in Poland (Supplementary File S1).

Before completing the questionnaire, each respondent received brief information on the definition of primary care. A primary care physician was defined as the doctor chosen by a patient within primary care services (pol. Podstawowa opieka zdrowotna; POZ)—typically a family medicine specialist, general practitioner, or internist for adults—serving as the initial point of contact and coordinating access to publicly financed services under the National Health Fund (NFZ). Care is delivered within the practice indicated in the patient’s enrollment declaration.

All respondents indicated whether they had visited a primary care physician (in person or via teleconsultation) for their own health in the preceding 12 months. A series of questions on lifestyle-related education and preventive services was addressed. Those who visited primary care physicians in the last 12 months were asked about preventive health services and health education offered to them when they visited the primary care clinic. Moreover, all respondents were asked about their perceptions of the opportunity to discuss preventive care with their primary care physician, as well as their willingness to receive an annual individualized prevention plan (screening tests and lifestyle recommendations to implement for healthy living) from the primary care physician.

A pilot study was conducted among 14 adults, who completed the questionnaire twice with a 7 day interval. Following this procedure, three items were revised to improve readability and consistency. Kappa coefficients for the critical questions ranged from 0.68 to 0.94.

### 2.3 Statistical analysis

Analyses were performed in IBM SPSS Statistics v29 (IBM Corp., Armonk, NY, United States). Categorical variables are presented as counts and percentages. Cross-tabulation with chi-square tests was used to compare differences between categorical variables.

For analysis, responses of “definitely yes” and “rather yes” were combined into a single affirmative category. The same approach was applied to responses “very good” and “good.” Multivariable logistic regression models were prepared to identify factors associated with the perception of preventive services offered in primary care clinics. In bivariable logistic regression, all variables were considered separately. Variables statistically significant in the bivariable model were included in the multivariable logistic regression model. The strengths of associations were reported as odds ratios (ORs) with 95% confidence intervals (CIs). Statistical significance was defined as *p* < 0.05.

## 3 Results

Completed questionnaires were received from 1,090 respondents, aged 18 years and over; 52.7% were females (Table 1). Among the respondents, 67.3% (*n* = 734) had visited a primary care physician due to their own health status at least once in the last 12 months. Detailed characteristics of the study population by visiting a primary care physician in the last 12 months is presented in Table 1.

**Table 1:** Characteristics of the study population by visiting primary care physician in the last 12 months (*n* = 1,090).

Variable	Overall		Visiting primary care physician in the last 12 months due to own health status				
			Yes (*n* = 734)		No (*n* = 356)		*p*
	*n*	%	*n*	%	*n*	%	
Gender							
Female	574	52.7	402	70.0	172	30.0	**0.04**
Male	516	47.3	332	64.3	184	35.7	
Age [years]							
18–29	130	11.9	81	62.3	49	37.7	**<0.001**
30–39	217	19.9	110	50.7	107	49.3	
40–49	213	19.5	127	59.6	86	40.4	
50–59	195	17.9	148	75.9	47	24.1	
60+	335	30.7	268	80.0	67	20.0	
Educational level							
Primary	16	1.5	6	37.5	10	62.5	**0.05**
Vocational	102	9.4	61	59.8	41	40.2	
Secondary	460	42.2	328	71.3	132	28.7	
Higher	512	47.0	339	66.2	173	33.8	
Marital status							
Single	287	26.3	174	60.6	113	39.4	**0.002**
Married	583	53.5	414	71.0	169	29.0	
Informal relationship	163	15.0	101	62.0	62	38.0	
Other	57	5.2	45	78.9	12	21.1	
Place of residence							
Rural area	409	37.5	270	66.0	139	34.0	0.9
City below 20,000 residents	140	12.8	96	68.6	44	31.4	
City from 20,000 to 99,999 residents	218	20.0	148	67.9	70	32.1	
City from 100,000 to 499,999 residents	181	16.6	125	69.1	56	30.9	
City ≥500,000 residents	142	13.0	95	66.9	47	33.1	
Having children under 18 years							
Yes	302	27.7	193	63.9	109	36.1	0.1
No	788	72.3	541	68.7	247	31.3	
Number of household members							
1 (living alone)	151	13.9	107	70.9	44	29.1	**0.04**
2	432	39.6	305	70.6	127	29.4	
3 or more	507	46.5	322	63.5	185	36.5	
Occupational status							
Active	671	61.6	420	62.6	251	37.4	**<0.001**
Passive	419	38.4	314	74.9	105	25.1	

Statistically significant values are bolded.

### 3.1 Preventive health services and health education offered to patients visiting primary care

Among those respondents who visited a primary care physician in the last 12 months (*n* = 734), 53% declared having received at least one healthy lifestyle-related advice from the primary care staff (physician, nurse, midwife, or administrative staff) (Table 2). Preventive tests appropriate for the patient’s age (33.2%) were the most common healthy lifestyle-related topic discussed by patients with primary care providers (Table 2). Moreover, 19.8% of respondents who visited primary care physicians in the last 12 months declared that primary care clinic staff discussed with them principles of healthy eating, 17.4% were advised on physical activity, and 16.8% received recommendations for a healthy lifestyle (Table 2). Methods to prevent overweight and obesity were discussed with 11.9% of patients who visited primary care practices. Less than 10% of respondents who visited primary care in the last 12 months discussed the health effects of tobacco use and methods for quitting or alcohol consumption (Table 2).

**Table 2:** Preventive health services and health education offered to patients who visited the primary care clinic at least once in the last 12 months (*n* = 734).

In the past 12 months, have the staff (i.e., primary care physician or nurse, midwife, or administrative staff) in your primary care clinic discussed with you the following healthy lifestyle-related topics?—positive answers “yes” (multiple-choice question)														
	Principles of healthy eating		Your physical activity		Recommendations for a healthy lifestyle		Preventive tests appropriate for your age		Methods to prevent overweight and obesity		Health effects of tobacco use and methods for quitting		Alcohol consumption	
Overall *n* (%)	145 (19.8%)		128 (17.4%)		123 (16.8%)		244 (33.2%)		87 (11.9%)		51 (6.9%)		36 (4.9%)	
Variable	%	*p*	%	*p*	%	*p*	%	*p*	%	*p*	%	*p*	%	*p*
Gender														
Female (*n* = 402)	17.9	0.2	12.7	**<0.001**	14.2	**0.04**	28.6	**0.003**	10.2	0.1	5.0	**0.02**	2.0	**<0.001**
Male (*n* = 332)	22.0		23.2		19.9		38.9		13.9		9.3		8.4	
Age [years]														
18–29 (*n* = 81)	25.9	0.2	29.6	**0.03**	12.3	0.5	23.5	**<0.001**	14.8	0.1	14.8	**0.04**	11.1	**0.005**
30–39 (*n* = 110)	14.5		13.6		20.0		13.6		10.0		5.5		2.7	
40–49 (*n* = 127)	20.5		15.7		16.5		31.5		12.6		6.3		8.7	
50–59 (*n* = 148)	23.0		14.2		14.2		34.5		16.9		8.1		4.1	
60+ (*n* = 268)	17.9		17.9		18.3		44.4		8.6		4.9		2.6	
Educational level														
Higher (*n* = 339)	19.2	0.7	17.1	0.8	13.6	**0.03**	28.3	**0.01**	10.6	0.3	7.1	0.9	5.9	0.2
Less than higher (*n* = 395)	20.3		17.7		19.5		37.5		12.9		6.8		4.1	
Marital status														
Single (*n* = 174)	23.0	0.6	17.2	0.7	12.6	0.4	29.9	0.8	13.2	0.9	8.0	0.3	7.5	0.3
Married (*n* = 414)	18.8		18.1		18.6		34.3		11.8		5.6		4.6	
Informal relationship (*n* = 101)	19.8		17.8		15.8		33.7		9.9		8.9		3.0	
Other (*n* = 45)	15.6		11.1		17.8		35.6		11.1		11.1		2.2	
Place of residence														
Rural area (*n* = 270)	18.1	0.2	15.9	0.2	15.2	0.2	30.0	0.5	11.5	0.8	6.3	0.8	3.3	0.1
City below 20,000 residents (*n* = 96)	28.1		21.9		13.5		31.3		13.5		5.2		6.3	
City from 20,000 to 99,999 residents (*n* = 148)	18.2		12.8		16.2		34.5		10.1		8.8		2.7	
City from 100,000 to 499,999 residents (*n* = 125)	20.8		21.6		24.0		38.4		14.4		7.2		8.8	
City ≥500,000 residents (*n* = 95)	16.8		18.9		15.8		35.8		10.5		7.4		6.3	
Having children under 18 years of age														
Yes (*n* = 193)	16.1	0.1	16.6	0.7	17.6	0.7	24.9	**0.004**	13.5	0.4	7.8	0.6	8.3	**0.01**
No (*n* = 541)	21.1		17.7		16.5		36.2		11.3		6.7		3.7	
Number of household members														
1 (living alone) (*n* = 107)	10.3	**0.01**	17.8	0.9	14.0	0.6	29.9	**<0.001**	7.5	0.3	4.7	0.1	4.7	0.2
2 (*n* = 305)	23.6		17.0		18.0		41.3		13.4		5.6		3.3	
3 or more (*n* = 322)	19.3		17.7		16.5		26.7		11.8		9.0		6.5	
Occupational status														
Active (*n* = 420)	20.2	0.7	20.0	**0.03**	17.9	0.4	29.5	**0.01**	12.1	0.8	8.3	0.09	7.4	**<0.001**
Passive (*n* = 314)	19.1		14.0		15.3		38.2		11.5		5.1		1.6	

Statistically significant values are bolded.

There were socio-demographic differences (mostly by gender and age) in the percentage of respondents who received healthy lifestyle-related advice from the primary care staff in the last 12 months (Table 2).

In the multivariable logistic regression model, female gender (compared to male; aOR: 1.72 [1.28–2.32], *p* < 0.001) and having higher education (compared to less than higher; aOR: 1.38 [1.03–1.86]; *p* < 0.03) were significantly associated with higher odds of lack of discussion on lifestyle-related topics between patients and primary care staff (Table 3).

**Table 3:** Factors associated with a declaration that the primary care staff did not discuss any lifestyle-related topics with the patient among those who visited primary care practice in the past 12 months (*n* = 734).

Factors associated with a declaration that the primary care staff did not discuss any lifestyle-related topics with the patient						
Variable	%	*p*	Bivariable logistic regression		Multivariable logistic regression	
			OR (95% CI)	*p*	aOR (95% CI)	*p*
Gender						
Female (*n* = 402)	53.2	**<0.001**	1.75 (1.30–2.35)	**<0.001**	1.72 (1.28–2.32)	**<0.001**
Male (*n* = 332)	39.5		Reference		Reference	
Age [years]						
18–29 (*n* = 81)	43.2	0.1	Reference			
30–39 (*n* = 110)	56.4		1.70 (0.95–3.03)	0.07		
40–49 (*n* = 127)	48.0		1.22 (0.69–2.13)	0.5		
50–59 (*n* = 148)	49.3		1.28 (0.74–2.21)	0.4		
60+ (*n* = 268)	42.5		0.97 (0.59–1.61)	0.9		
Educational level						
Higher (*n* = 339)	51.6	**0.02**	1.41 (1.06–1.89)	**0.02**	1.38 (1.03–1.86)	**0.03**
Less than higher (*n* = 395)	43.0		Reference		Reference	
Marital status						
Single (*n* = 174)	47.7	0.8	Reference			
Married (*n* = 414)	47.1		0.98 (0.69–1.39)	0.9		
Informal relationship (*n* = 101)	43.6		0.85 (0.52–1.39)	0.5		
Other (*n* = 45)	51.1		1.15 (0.60–2.21)	0.7		
Place of residence						
Rural area (*n* = 270)	51.5	0.4	1.23 (0.77–1.97)	0.4		
City below 20,000 residents (*n* = 96)	42.7		0.86 (0.49–1.53)	0.6		
City from 20,000 to 99,999 residents (*n* = 148)	46.6		1.01 (0.60–1.70)	0.9		
City from 100,000 to 499,999 residents (*n* = 125)	41.6		0.83 (0.48–1.41)	0.5		
City ≥500,000 residents (*n* = 95)	46.3		Reference			
Having children under 18 years of age						
Yes (*n* = 193)	49.2	0.5	1.13 (0.81–1.57)	0.5		
No (*n* = 541)	46.2		Reference			
Number of household members						
1 (living alone) (*n* = 107)	52.3	0.2	1.17 (0.75–1.81)	0.5		
2 (*n* = 305)	43.6		0.82 (0.60–1.13)	0.2		
3 or more (*n* = 322)	48.4		Reference			
Occupational status						
Active (*n* = 420)	47.9	0.6	1.08 (0.81–1.45)	0.6		
Passive (*n* = 314)	45.9		Reference			

Statistically significant values are bolded.

### 3.2 Public perception and attitudes towards preventive health services offered by primary care physicians

Among all respondents (*n* = 1,090), 27.9% rated the opportunity to discuss preventive care with their primary care physician as very good, and 38.3% rated this opportunity as good (Table 4). Moreover, 67.8% of respondents (37.5%—rather yes and 30.3%—definitely yes) declared a willingness to receive an annual individualized prevention plan (screening tests and lifestyle recommendations to implement for healthy living) from their primary care physician. Details are presented in Table 4.

**Table 4:** Public perception and attitudes towards preventive health services offered by primary care physicians in a general population of adults in Poland (*n* = 1,090).

Public perception and attitudes towards preventive health services offered by primary care physicians		
How to you rate the opportunity to discuss preventive care with your primary care physician?		
Very good	304	27.9
Good	417	38.3
Fair	226	20.7
Bad	48	4.4
Very bad	14	1.3
I do not know/no opinion	81	7.4
Would you like your primary care physician to set an annual individualized prevention plan (screening tests and lifestyle recommendations to implement for healthy living) with you?		
Definitely no	27	2.5
Rather no	100	9.2
Rather yes	409	37.5
Definitely yes	330	30.3
I do not know/difficult to tell	224	20.6

In the multivariable logistic regression, male gender (compared to female; aOR: 1.40 [1.08–1.82]; *p* = 0.01) and visiting primary care physician (at least once) in the last 12 months (aOR: 2.32 [1.76–3.05]; *p* < 0.001) were significantly associated with higher odds of good perception of the opportunity to discuss preventive care with a primary care physician among adults in Poland (Table 5).

**Table 5:** Factors associated with a good perception of the opportunity to discuss preventive care with a primary care physician among adults in Poland (*n* = 1,090).

How to you rate the opportunity to discuss preventive care with your primary care physician?—“very good” or “good”						
Variable	%	*p*	Bivariable logistic regression		Multivariable logistic regression	
			OR (95% CI)	*p*	aOR (95% CI)	*p*
Gender						
Female (*n* = 574)	63.4	**0.04**	Reference	0.04	Reference	**0.01**
Male (*n* = 516)	69.2		1.30 (1.01–1.67)		1.40 (1.08–1.82)	
Age [years]						
18–29 (*n* = 130)	61.5	**0.03**	Reference		Reference	
30–39 (*n* = 217)	60.4		0.95 (0.61–1.49)	0.8	0.96 (0.61–1.54)	0.9
40–49 (*n* = 213)	63.4		1.08 (0.69–1.70)	0.7	0.99 (0.61–1.59)	0.9
50–59 (*n* = 195)	68.7		1.37 (0.86–2.19)	0.2	1.07 (0.65–1.77)	0.8
60+ (*n* = 335)	71.9		1.60 (1.05–2.45)	**0.03**	1.24 (0.77–1.97)	0.4
Educational level						
Higher (*n* = 512)	66.8	0.7	1.06 (0.82–1.36)	0.7		
Less than higher (*n* = 578)	65.6		Reference			
Marital status						
Single (*n* = 287)	59.6	**0.04**	Reference		Reference	
Married (*n* = 583)	69.3		1.53 (1.14–2.06)	**0.01**	1.36 (0.99–1.86)	0.06
Informal relationship (*n* = 163)	65.0		1.26 (0.85–1.88)	0.3	1.28 (0.85–1.92)	0.2
Other (*n* = 57)	70.2		1.60 (0.86–2.95)	0.2	1.34 (0.70–2.56)	0.4
Place of residence						
Rural area (*n* = 409)	68.5	0.6	1.04 (0.69–1.57)	0.9		
City below 20,000 residents (*n* = 140)	64.3		0.86 (0.53–1.41)	0.6		
City from 20,000 to 99,999 residents (*n* = 218)	65.6		0.91 (0.58–1.43)	0.7		
City from 100,000 to 499,999 residents (*n* = 181)	61.9		0.78 (0.49–1.24)	0.3		
City ≥500,000 residents (*n* = 142)	67.6		Reference			
Having children under 18 years of age						
Yes (*n* = 302)	63.9	0.3	0.87 (0.66–1.15)	0.3		
No (*n* = 788)	67.0		Reference			
Number of household members						
1 (living alone) (*n* = 151)	60.9	0.3	Reference			
2 (*n* = 432)	67.8		1.35 (0.92–1.99)	0.1		
3 or more (*n* = 507)	66.3		1.26 (0.87–1.83)	0.2		
Occupational status						
Active (*n* = 671)	64.7	0.2	0.84 (0.65–1.09)	0.2		
Passive (*n* = 419)	68.5		Reference			
Visiting primary care physician (at least once) in the last 12 months						
Yes (*n* = 734)	72.8	**<0.001**	2.41 (1.85–3.14)	**<0.001**	2.32 (1.76–3.05)	**<0.001**
No (*n* = 356)	52.5		Reference		Reference	

Statistically significant values are bolded.

In the multivariable logistic regression, being married (compared to being single; aOR: 1.46 [1.07–1.98]; *p* = 0.02), and visiting primary care physician (at least once) in the last 12 months (aOR: 2.66 [2.03–3.49]; *p* < 0.001) were significantly associated with willingness to receive an annual individualized prevention plan from the primary care physician (Table 6).

**Table 6:** Factors associated with willingness to receive an annual individualized prevention plan from the primary care physician (*n* = 1,090).

Would you like your primary care physician to set an annual individualized prevention plan (screening tests and lifestyle recommendations to implement for healthy living) with you?—“definitely yes” or “rather yes”						
Variable	%	*p*	Bivariable logistic regression		Multivariable logistic regression	
			OR (95% CI)	*p*	aOR (95% CI)	*p*
Gender						
Female (*n* = 574)	70.7	**0.03**	1.33 (1.03–1.71)	**0.03**	1.28 (0.99–1.67)	0.06
Male (*n* = 516)	64.5		Reference		Reference	
Age [years]						
18–29 (*n* = 130)	66.9	0.7	Reference			
30–39 (*n* = 217)	64.1		0.88 (0.56–1.39)	0.6		
40–49 (*n* = 213)	68.1		1.05 (0.66–1.68)	0.8		
50–59 (*n* = 195)	68.2		1.06 (0.66–1.70)	0.8		
60+ (*n* = 335)	70.1		1.16 (0.75–1.79)	0.5		
Educational level						
Higher (*n* = 512)	69.9	0.2	1.20 (0.93–1.55)	0.2		
Less than higher (*n* = 578)	65.9		Reference			
Marital status						
Single (*n* = 287)	60.6	**0.02**	Reference		Reference	
Married (*n* = 583)	70.8		1.58 (1.17–2.12)	**0.003**	1.46 (1.07–1.98)	**0.02**
Informal relationship (*n* = 163)	69.3		1.47 (0.98–2.21)	0.07	1.48 (0.97–2.25)	0.07
Other (*n* = 57)	68.4		1.41 (0.77–2.58)	0.3	1.11 (0.59–2.08)	0.7
Place of residence						
Rural area (*n* = 409)	65.8	0.3	Reference			
City below 20,000 residents (*n* = 140)	65.0		0.97 (0.65–1.45)	0.9		
City from 20,000 to 99,999 residents (*n* = 218)	72.0		1.34 (0.94–1.92)	0.1		
City from 100,000 to 499,999 residents (*n* = 181)	65.7		0.99 (0.69–1.44)	0.9		
City ≥500,000 residents (*n* = 142)	72.5		1.38 (0.90–2.10)	0.1		
Having children under 18 years of age						
Yes (*n* = 302)	69.9	0.4	1.14 (0.86–1.52)	0.4		
No (*n* = 788)	67.0		Reference			
Number of household members						
1 (living alone) (*n* = 151)	60.9	0.1	Reference			
2 (*n* = 432)	69.4		1.46 (0.99–2.14)	0.06		
3 or more (*n* = 507)	68.4		1.39 (0.95–2.03)	0.09		
Occupational status						
Active (*n* = 671)	68.1	0.8	1.04 (0.80–1.35)	0.8		
Passive (*n* = 419)	67.3		Reference			
Visiting primary care physician (at least once) in the last 12 months						
Yes (*n* = 734)	75.2	**<0.001**	2.74 (2.10–3.58)	**<0.001**	2.66 (2.03–3.49)	**<0.001**
No (*n* = 356)	52.5		Reference		Reference	

Statistically significant values are bolded.

## 4 Discussion

This nationwide cross-sectional study provided comprehensive characteristics of public perception and expectations towards lifestyle-related education and preventive services in primary care in Poland. Only half of the respondents who visited primary care in the last 12 months declared having received at least one healthy lifestyle-related advice from the primary care staff. Preventive tests appropriate for the patient’s age were the most common preventive service offered in primary care (one-third of respondents), followed by education on principles of healthy eating and physical activity. Females, as well as those with higher education, were identified as socio-demographic groups, often omitted in preventive actions offered by primary care staff. In a general, nationwide sample of adults in Poland, over two-thirds rated the opportunity to discuss preventive care with their primary care physician and declared willingness to receive an annual individualized prevention plan. Marital status and history of primary care services utilization were the only factors significantly associated with personal willingness to receive an annual individualized prevention plan.

Primary care is often a patient’s first line of contact with healthcare (3, 7). Family doctors are often the ones who know the patient the longest and best. In Poland, there is a high level of public trust in family doctors (23). In Poland, preventive primary care services are available and publicly funded (1, 3, 17). The extent to which primary care practice staff engage in health education and primary services depends on the practice. Findings from this study showed that only half (53%) of those who visited primary care in the last 12 months had received at least one healthy lifestyle-related advice or preventive service from the primary care staff (physician, nurse, midwife, or administrative staff). Previous research from European countries indicates that approximately 50%–70% of patients report receiving advice on lifestyle-related behaviors such as diet, physical activity, smoking cessation, or alcohol consumption during primary care consultations (24–26). Although the reported prevalence varies depending on the healthcare system and the type of counseling assessed, these findings suggest that preventive counseling remains inconsistently implemented across European primary care systems. This finding underlines an insufficient level of public health actions undertaken by primary care providers in Poland. Further analyses carried out among primary care providers are needed to identify potential barriers in the implementation of lifestyle-related education and preventive services in primary care. We can hypothesize that workload and lack of time may be the main factors contributing to the limited implementation of public health actions by primary care providers (27).

Preventive tests appropriate for the patient’s age (33.2%) were the most common healthy lifestyle-related topic discussed by patients with primary care providers. There are three publicly funded population-based cancer screenings (mammography, colonoscopy, and cytology) offered for different age groups in Poland. Moreover, a primary care physician can order a variety of laboratory tests (with morphology as the most common one), both as part of diagnostic and treatment, as well as screening check-ups. Except that, during the study period, there were dedicated preventive programs like Prevention 40+ (ended up in April 2025), and My Health (started in May 2025) (28). Less than one-fifth of patients who visited primary care practitioners received education on principles of healthy eating (19.8%) or physical activity (17.4%). Diet and physical activity are significant lifestyle factors for most of the non-communicable diseases (29, 30). There was also low engagement of primary care physicians in the education on alcohol and tobacco prevention, as well as education on obesity and weight management. This observation suggests an urgent need to increase primary care providers’ involvement in education on healthy lifestyle and principles of lifestyle medicine (29–31).

Findings from this study also revealed socio-demographic inequalities in the receipt of healthy lifestyle-related advice and preventive services by primary care patients. Females and those with higher education were less likely to receive lifestyle-related education and preventive services in primary care. These two groups are often considered in Poland as those with higher health literacy levels (32, 33). We can hypothesize that observed differences between female respondents and males, as well as between individuals with different educational levels, may reflect variations in health literacy, patient expectations, and communication patterns in primary care consultations. Previous studies have shown that individuals with higher education levels often report higher expectations regarding preventive services and may be more critical when evaluating the quality of preventive counseling provided by healthcare professionals (34). In addition, gender differences in patient–physician communication and preventive care utilization have been reported in several studies, suggesting that women and men may perceive preventive counseling differently (35).

Health communication and the communication skills of medical personnel play an important role in building awareness and activating patients to use preventive services (36, 37). In this study, 66.2% rated the opportunity to discuss preventive care with their primary care physician as very good or good. This observation suggests further needs in the implementation of education and training of healthcare professionals on health communication. Motivational interviewing and plain language should be considered as key elements of health communication on preventive services (37, 38). Males were more likely than females to highly rate the opportunity to discuss preventive care with their primary care physician. This observation may result from the fact that preventive services for females, including breast and cervical cancer prevention, are more complicated and require more time for comprehensive explanations.

One of the key criteria for effective prevention is performing the right tests at the right time. For this reason, expert groups and international organizations are developing guidelines and recommendations for performing preventive testing in specific age groups (39). In this study, respondents were asked about the potential new preventive service in primary care—an annual individualized prevention plan from the primary care physician. Findings from this study showed that 67.8% of respondents declared a willingness to receive an annual individualized prevention plan (screening tests and lifestyle recommendations to implement for healthy living) from their primary care physician. This observation revealed relatively high social interest in the personalized prevention services in primary care (39). Those who were married were more likely to declare willingness to receive this kind of preventive service.

Personal experience with primary care and utilization of primary care services—visiting a primary care physician (at least once) in the last 12 months—was also significantly associated with higher rates of opportunity to discuss preventive care with their primary care physician, as well as willingness to receive an annual individualized prevention plan. This observation shows how personal experiences shape public expectations towards healthcare services.

In this study, age, place of residence, and economic status were not significantly associated with attitudes toward preventive services, suggesting that expectations for preventive care were relatively consistent across demographic groups within this sample.

### 4.1 Practical implications

Findings from this study underline the urgent need to improve the lifestyle-related education and preventive services in primary care in Poland. Only half of those who visited primary care in the last 12 months had received lifestyle-related education and preventive services. Educational and organizational activities are needed to strengthen the involvement of primary care staff in public health activities. Moreover, this study revealed that females and those with higher education are less likely to receive preventive services in primary care, which suggests that educational activities targeted to primary care staff should address these socio-demographic gaps in preventive practices in primary care. This study also suggest that there is a high social acceptance for the implementation of an annual individualized prevention plan (screening tests and lifestyle recommendations to implement for healthy living). Policymakers may consider introducing dedicated billing codes or financial incentives within the National Health Fund to support the implementation of individualized prevention plans in primary care. Further educational campaigns are also needed to strengthen the awareness of preventive services available in primary care among the general population.

### 4.2 Limitations

This is a cross-sectional survey carried out using the CAWI technique, so those without Internet access [4% of households in Poland (22)] could not participate in this study. The study was carried out in a general, nationwide sample of adults in Poland, and only 67.3% of respondents used primary care services in the last 12 months, prior to the study. Further studies should be carried out among particular subgroups of patients who use primary care services. Data were self-declared. Because respondents were asked to recall preventive counseling received during the previous 12 months, recall bias cannot be excluded. Medical documentation was not screened due to data protection reasons. To minimize respondent burden and survey fatigue, the length of the questionnaire was intentionally restricted, which limited the scope of preventive services assessed. Selection of questions, especially the questions on lifestyle-related education and preventive services offered by the primary care provider, was based on a literature review, and further studies may assess a wider scope of preventive services.

## 5 Conclusion

This study showed a low level of implementation of lifestyle-related education and preventive services in primary care in Poland. Preventive tests appropriate for the patient’s age were the most common preventive services offered in primary care. Females and those with higher education were identified as sociodemographic groups that are overlooked in educational and preventive activities in primary care. Over two-thirds of adults in Poland expressed a willingness to receive an annual individualized prevention plan from their primary care physician, indicating further needs in the development of preventive services in primary care. These findings suggest that demand for preventive services in primary care appears relatively widespread across demographic groups.

## Data Availability

The raw data supporting the conclusions of this article will be made available by the authors, without undue reservation.

## References

[1] Sowada C Sagan A Kowalska-Bobko I Badora-Musial K Bochenek T Domagala A . Poland: health system review. Health Syst Transit. (2019) 21:1–234. 31333192

[2] PHC Act of 27 October 2017 on primary health care. J Laws Republic Poland. (2017) item 2217.

[3] Kringos D Boerma W Bourgueil Y Cartier T Dedeu T Hasvold T . The strength of primary care in Europe: an international comparative study. Br J Gen Pract. (2013) 63:e742–50. doi: 10.3399/bjgp13X674422, 24267857 PMC3809427

[4] Li J Pandian V Davidson PM Song Y Chen N Fong DYT. Burden and attributable risk factors of non-communicable diseases and subtypes in 204 countries and territories, 1990-2021: a systematic analysis for the global burden of disease study 2021. Int J Surg. (2025) 111:2385–97. doi: 10.1097/JS9.0000000000002260, 39869379 PMC12372739

[5] Uchmanowicz I Czapla M Lomper K Iovino P Rosiek-Biegus M Surma S . Effective implementation of preventive cardiology guidelines: pathways to success. Eur J Prev Cardiol. (2025):zwaf448. doi: 10.1093/eurjpc/zwaf448 40680090

[6] Beaglehole R Bonita R Horton R Adams C Alleyne G Asaria P . Priority actions for the non-communicable disease crisis. Lancet. (2011) 377:1438–47. doi: 10.1016/S0140-6736(11)60393-0, 21474174

[7] Starfield B Shi L Macinko J. Contribution of primary care to health systems and health. Milbank Q. (2005) 83:457–502. doi: 10.1111/j.1468-0009.2005.00409.x, 16202000 PMC2690145

[8] Macinko J Starfield B Erinosho T. The impact of primary healthcare on population health in low- and middle-income countries. J Ambul Care Manage. (2009) 32:150–71. doi: 10.1097/JAC.0b013e3181994221, 19305227

[9] Kringos DS Boerma WGW van der Zee J Groenewegen PP. Europe’s strong primary care systems are linked to better population health. Health Aff (Millwood). (2013) 32:686–94. doi: 10.1377/hlthaff.2012.1242, 23569048

[10] Wagner EH Austin BT Von Korff M. Organizing care for patients with chronic illness. Milbank Q. (1996) 74:511–44. doi: 10.2307/3350391 8941260

[11] Agrawal S Gołębiowska J Makuch S Mazur G. Prevalence of use of preventive services in Poland: result from a population-based nationwide study. J Clin Med. (2021) 10:2084. doi: 10.3390/jcm10102084, 34066239 PMC8150860

[12] Marcinowicz L Konstantynowicz J Chlabicz S. The patient’s view of the acceptability of the primary care in Poland. Int J Qual Health Care. (2008) 20:277–83. doi: 10.1093/intqhc/mzn020, 18492707

[13] Krztoń-Królewiecka A Oleszczyk M Windak A. Do polish primary care physicians meet the expectations of their patients? An analysis of polish QUALICOPC data. BMC Fam Pract. (2020) 21:118. doi: 10.1186/s12875-020-01190-1, 32576153 PMC7313208

[14] Brotons C Björkelund C Bulc M Ciurana R Godycki-Cwirko M Jurgova E . Prevention and health promotion in clinical practice: the views of general practitioners in Europe. Prev Med. (2005) 40:595–601. doi: 10.1016/j.ypmed.2004.07.020, 15749144

[15] Ministry of Health. Prevention reinvented – the my health program. (2025). Available online at: https://www.gov.pl/web/zdrowie/profilaktyka-na-nowo-czyli-program-moje-zdrowie (Accessed December 30, 2025)

[16] Frenk J Chen L Bhutta ZA Cohen J Crisp N Evans T . Health professionals for a new century: transforming education to strengthen health systems. Lancet. (2010) 376:1923–58. doi: 10.1016/S0140-6736(10)61854-5 21112623

[17] Zakrzewska K Parda N Rosińska M. Prevention programmes in primary health care – 8 aspects of their effective implementation. Przegl Epidemiol. (2017) 71:259–69. 28872291

[18] Nationwide Research Panel Ariadna. About us. (2025). Available online at: https://panelariadna.com/ (Accessed December 30, 2025).

[19] Olearczyk A Sękowski K Jankowski M Moczeniat G Kamińska A Grudziąż-Sękowska J. Prevalence and drivers of missed healthcare appointments in Poland: insights from a 2025 survey. Med Sci Monit. (2026) 32:e951944. doi: 10.12659/MSM.951944 41764069 PMC12961910

[20] Kamińska A Pinkas J Jankowski M. Factors associated with the frequency of eye examinations among adults in Poland – a nationwide cross-sectional survey, December 2022. Ann Agric Environ Med. (2023) 30:287–95. doi: 10.26444/aaem/159152 37387379

[21] Sękowski K Mazurek A Grześczyk-Nojszewska Z Jankowski M Kamińska A Olearczyk A . Public awareness of obesity causes, complications, and treatment methods in a representative sample of adults in Poland. Front Public Health. (2025) 13:1656877. doi: 10.3389/fpubh.2025.1656877, 41048255 PMC12488597

[22] Statistics Poland. Information society in Poland 2024 [internet]. (2024). Available online at: https://stat.gov.pl/files/gfx/portalinformacyjny/en/defaultaktualnosci/3417/2/14/1/information_society_in_poland_2024.pdf (Accessed December 29, 2025).

[23] Pogorzelska K Marcinowicz L Chlabicz S. Understanding satisfaction and dissatisfaction of patients with telemedicine during the COVID-19 pandemic: an exploratory qualitative study in primary care. PLoS One. (2023) 18:e0293089. doi: 10.1371/journal.pone.0293089, 37847684 PMC10581451

[24] Brotons C Bulc M Sammut MR Sheehan M Manuel da Silva Martins C Björkelund C . Attitudes toward preventive services and lifestyle: the views of primary care patients in Europe. The EUROPREVIEW patient study. Fam Pract. (2012) 29:i168–76. doi: 10.1093/fampra/cmr102 22399549

[25] Noordman J Koopmans B Korevaar JC van der Weijden T van Dulmen S. Exploring lifestyle counselling in routine primary care consultations: the professionals’ role. Fam Pract. (2013) 30:332–40. doi: 10.1093/fampra/cms077 23221102

[26] Kiestra L de Vries IAC Mulder BC. Determinants of lifestyle counseling and current practices: a cross-sectional study among Dutch general practitioners. PLoS One. (2020) 15:e0235968. doi: 10.1371/journal.pone.0235968 32692740 PMC7373284

[27] Russo G Perelman J Zapata T Šantrić-Milićević M. The layered crisis of the primary care medical workforce in the European region: what evidence do we need to identify causes and solutions? Hum Resour Health. (2023) 21:55. doi: 10.1186/s12960-023-00842-4, 37443059 PMC10347862

[28] Sierpiński R Kilanowski I. Descriptive analysis of the implementation of the program “prevention 40 PLUS” in Poland, July 2021–December 2024. Eur J Health Policy Hum Care Med Ethics. (2025) 4:55–64. doi: 10.21697/ejhp.0106.05

[29] Santos L. The impact of nutrition and lifestyle modification on health. Eur J Intern Med. (2022) 97:18–25. doi: 10.1016/j.ejim.2021.09.020, 34670680

[30] Marquez DX Aguiñaga S Vásquez PM Conroy DE Erickson KI Hillman C . Physical activity and quality of life and well-being: a systematic review. Transl Behav Med. (2020) 10:1098–109. doi: 10.1093/tbm/ibz198 33044541 PMC7752999

[31] Bharati R Kovach KA Bonnet JP Sayess P Polk E Harvey K . Incorporating lifestyle medicine into primary care practice: perceptions and practices of family physicians. Am J Lifestyle Med. (2023) 17:704–16. doi: 10.1177/15598276211072506, 37711349 PMC10498979

[32] Lopuszanska-Dawid M. Trends in health behavior of polish women in 1986-2021: the importance of socioeconomic status. Int J Environ Res Public Health. (2023) 20:3964. doi: 10.3390/ijerph20053964, 36900975 PMC10001600

[33] Cutler DM Lleras-Muney A. Understanding differences in health behaviors by education. J Health Econ. (2010) 29:1–28. doi: 10.1016/j.jhealeco.2009.10.003, 19963292 PMC2824018

[34] Coughlin SS Vernon M Hatzigeorgiou C George V. Health literacy, social determinants of health, and disease prevention and control. J Environ Health Sci. (2020) 6:3061. doi: 10.15436/2378-6843.20.1865, 33604453 PMC7889072

[35] Diehl K Gansefort D Herr RM Görig T Bock C Mayer M . Physician gender and lifestyle counselling to prevent cardiovascular disease: a nationwide representative study. J Public Health Res. (2015) 4:534. doi: 10.4081/jphr.2015.534 26425495 PMC4568424

[36] Guo Z Wu Q Wang X Dai Y Ma Y Qiu Y . Effects of message framing and risk perception on health communication for optimum cardiovascular disease primary prevention: a protocol for a multicenter randomized controlled study. Front Public Health. (2024) 12:1308745. doi: 10.3389/fpubh.2024.1308745, 38550324 PMC10972929

[37] Srivastava SB. Language: a powerful tool in promoting healthy behaviors. Am J Lifestyle Med. (2019) 13:359–61. doi: 10.1177/1559827619839995, 31285717 PMC6600624

[38] Bahri AA. Motivational interviewing to promote healthy lifestyle behaviors: evidence, implementation, and digital applications. J Multidiscip Healthc. (2025) 18:6629–42. doi: 10.2147/JMDH.S557957, 41104302 PMC12526391

[39] Farina S Osti T Russo L Maio A Scarsi N Savoia C . The current landscape of personalised preventive approaches for non-communicable diseases: a scoping review. PLoS One. (2025) 20:e0317379. doi: 10.1371/journal.pone.0317379, 39804869 PMC11729939

