# Supplementary material for: Population-level evidence on lifestyle-related education and preventive services in primary care in Poland—a 2025 cross-sectional survey
Source: Front Public Health. 2026 Mar 27;14:1779567. doi: 10.3389/fpubh.2026.1779567 (PMC13066236; doi:10.3389/fpubh.2026.1779567)
Supplement: Supplementary file 1 [file Table_1.DOCX]

**Q1. Which types of physicians have you visited at least once in the past 12 months because of your health?**
*Select all that apply.*

1. Family physician / primary care physician (PCP; publicly funded)
2. Pediatrician
3. Medical specialist
4. Other (please specify): __________
5. I did not visit any physician in the past 12 months

**Q2. For what reasons did you visit your family physician/primary care physician in the past 12 months?**
*Select all that apply.* *[multiple responses allowed]*

1. An urgent visit for a cold, influenza, or another acute infection
2. A follow-up visit for a chronic condition (e.g., diabetes, hypertension, or another long-term illness)
3. An urgent visit due to a sudden deterioration in health (e.g., shortness of breath, chest pain, severe abdominal pain, or other acute symptoms)
4. A visit for mental health concerns (e.g., low mood/depression, anxiety, or sleep problems)
5. To obtain a referral to a medical specialist
6. To obtain an order/referral for diagnostic and/or laboratory tests
7. To obtain a prescription for medications
8. To obtain a medical leave certificate (“sick note”)
9. To obtain a certificate/attestation (e.g., for employment)
10. A preventive/health-check visit to review health status and complete recommended screening tests
11. A visit for vaccination (e.g., influenza)
12. Another reason (please specify): __________ *[do not randomize]*

**Q3. How long do you usually wait for an appointment with your family physician/primary care physician when you need to schedule one?**
*Select one.*

1. Same day
2. Next day
3. In 2–3 days
4. In 4–5 days
5. In 6–7 days
6. More than 7 days

**Q4. How do you usually schedule appointments with your family physician/primary care physician?**
*Select all that apply.* *[ multiple responses allowed]*

1. By telephone
2. By SMS/text message
3. Through the Internetowe Konto Pacjenta (IKP) patient portal
4. Through the clinic’s mobile app or website
5. In person at the clinic
6. Other (please specify): __________ *[do not randomize]*

**Q5. Do you trust your family physician/primary care physician?**
*Select one.*

1. definitely yes
2. rather yes
3. rather no
4. definitely no
5. I do not know/ difficult to tell

**Q6. Overall, how satisfied are you with the quality of consultations and treatment you receive in primary care?**
*Select one.*

1. very satisfied
2. somewhat satisfied
3. somewhat dissatisfied
4. very dissatisfied
5. I don’t know / difficult to tell

**Q7. How do you rate the performance of the following elements of the primary care system (i.e., your family physician/PCP)?**
*Please select one response for each item.*
*Response scale (horizontal): Very poor – Poor – Fair – Good – Very good – Don’t know/No opinion.*
Waiting time for an appointment with your family physician/PCP

- Distance from your home to the family physician/PCP clinic
- Courtesy of clinic staff at the family physician/PCP clinic
- Medical competence and knowledge of your family physician/PCP
- Your family physician’s/PCP’s engagement in solving your health problem
- Coordination of your care by the family physician/PCP and clinic staff (e.g., providing information on where and how to obtain further treatment)
- Willingness of your family physician/PCP to issue a referral to a specialist
- Willingness of your family physician/PCP to order diagnostic tests
- Opportunity to discuss preventive care with your family physician/PCP

**Q8. Did your family physician/primary care physician provide information about your health and treatment that was sufficient and easy to understand?**
*Select one.*

1. definitely yes
2. rather yes
3. rather no
4. definitely no
5. I do not know/ difficult to tell

**Q9. In the past 12 months, which of the following options were you informed about by staff at your primary care clinic (i.e., your family physician/PCP, nurse, midwife, or administrative staff)?**
*Select all that apply. [multiple responses allowed]*

1. Influenza vaccination
2. Vaccination against pneumococcal disease, RSV, or shingles
3. Participation in the Moje Zdrowie program (My Health)
4. Participation in the cardiovascular disease prevention program (CHUK)
5. Free mammography
6. Free cervical cytology (Pap test)
7. None of the above *[do not randomize]*

**Q10. In the past 12 months, which topics did staff at your primary care clinic (i.e., your family physician/PCP, nurse, midwife, or administrative staff) discuss with you?**
*Select all that apply.* *[; multiple responses allowed]*

1. Health effects of tobacco use and methods for quitting
2. Principles of healthy eating
3. Your physical activity
4. Alcohol consumption
5. Recommendations for a healthy lifestyle
6. Preventive tests appropriate for your age
7. Methods to prevent overweight and obesity
8. None of the above *[do not randomize]*

**Q11. Would you like your family physician/primary care physician to set an annual individualized prevention plan with you—that is, a plan of screening tests and lifestyle recommendations to implement for healthy living?**
*Select one.*

1. Definitely no
2. Rather no
3. Rather yes
4. Definitely yes
5. Don’t know / Not sure

**Q12. In your opinion, what are the most important problems in primary care (i.e., at your family physician/PCP clinic)?**
*Select all that apply.* *[randomize items; multiple responses allowed]*

1. Long waiting time for appointments
2. Lower staff engagement and poorer quality of services compared with private care
3. Insufficient number of medical personnel
4. Poorer access to information about one’s health status than in private care
5. Cost-saving on diagnostics and lack of referrals to specialists
6. Poor management of the healthcare system (inefficient organization of work) and inappropriate use of financial resources
7. Variation in quality of care across facilities in the country
8. Other (please specify): __________ *[do not randomize]*
